# Palliative Care in Congenital Syndrome of the Zika Virus Associated with Hospitalization and Emergency Consultation: Palliative Care and Congenital Syndrome of Zika

**DOI:** 10.1155/2018/1025193

**Published:** 2018-10-11

**Authors:** Aline Maria de Oliveira Rocha, Maria Julia Gonçalves de Mello, Juliane Roberta Dias Torres, Natalia de Oliveira Valença, Alessandra Costa de Azevedo Maia, Nara Vasconcelos Cavalcanti

**Affiliations:** ^1^Institute of Integral Medicine Prof. Fernando Figueira, Brazil; ^2^Institute of Integral Medicine Prof. Fernando Figueira, Universidade de Pernambuco, Brazil

## Abstract

**Background:**

Congenital syndrome of Zika virus (CSZV) is associated with neuromotor and cognitive developmental disorders, limiting the independence and autonomy of affected children and high susceptibility to complications, so palliative care needs to be discussed and applied.

**Aim:**

To identify factors associated with emergency visits and hospitalizations of patients with CSZV and clinical interventions performed from the perspective of palliative care.

**Design:**

This is a cross-sectional study with bidirectional longitudinal component. Data were collected between May and October 2017 through the review of medical records and interviews with relatives of patients hospitalized.

**Setting/Participants:**

The study was developed in a tertiary care hospital involving patients with confirmed CSZV born as of August 2015 and followed up until October 2017. Patients under investigation were excluded.

**Results:**

145 patients were followed up at the specialized outpatient clinic, 92 (63.5%) were consulted at least once in the emergency room, and 49% had already been hospitalized, with the main reason being neurological causes, while 24.1% had never required any emergency visit or hospitalization. No risk factors were associated with the occurrence of consultations or hospitalizations. Such events happened at an early age and were accompanied by a high number of invasive procedures and interventions. An approach in palliative care was only identified in two hospitalized patients.

**Conclusions:**

For the patient with known severe malformations caused by congenital infection by the Zika virus with indication of palliative care, this approach could be used in order to allow life without suffering and disproportionate invasive method.

## 1. Introduction

Between August and November 2015, a substantial increase in the number of cases of newborns with microcephaly [1], characterized by the World Health Organization (WHO) as an “anomaly in which the head circumference (HC) is less than two or more standard deviations than the reference for sex, age or gestational age” [2] and whose main causes are genetic or environmental reasons, such as radiation, drugs, fetal alcohol syndrome, and infections [3]. This clinical condition leads to medicalization, hospitalization, and invasive procedures such as ventriculoperitoneal shunt (VPS) and gastrostomy. The innumerable associated morbidities and limited quality of life emphasize the need to discuss palliative care in this group [4–6].

The prevalence of congenital microcephaly in Brazil was estimated at 1.98 per 10,000 live births by October 2015, a number that increased 3 to 12 times during the microcephaly outbreak, depending on the region considered [7, 8]. The majority of these patients were concentrated in the state of Pernambuco [9, 10].

It was possible to correlate such an aggravation to the infection by Zika virus during pregnancy, which is transmitted to the fetus and may result in miscarriage, fetal death, or congenital anomalies in several organs, being characterized as Congenital Syndrome of Zika virus (CSZV) [11–15]. CSZV has become a clinical condition associated with several complications, with a disabling and noncurable nature [16–21]. Among the malformations described in the literature are cerebral atrophy, absence of brain spins, craniofacial disproportion, cerebral calcifications, corpus callosum and cerebellar vermis dysgenesis, limb contractures, arthrogryposis, and auditory and ocular abnormalities [7, 17, 18].

Such clinical manifestations have caused from the first semester of life, for example, a high frequency of epileptic seizures, changes in tone, posture and mobility, irritability, intracranial hypertension secondary to hydrocephalus, and swallowing problems with greater predisposition to gagging and respiratory infections [19–21].

Palliative care is understood as active and total approach to care, which begins from diagnosis of life-limiting or life-threatening conditions and continues throughout the child's life and death [6]. Therefore, this type of care is clearly indicated for diseases with severe and incapacitating neurological manifestations, such as CSZV.

In pediatrics it should be considered for “complex chronic clinical conditions”, defined as situations that present at least 12 months of survival and affect one or more organ systems that require specialized pediatric care [4, 5]. What is aimed is the best quality of life for the patient and his family, attending to the physical, psychological, spiritual, and social needs.

Indications for palliative care in pediatrics may be represented in different groups defined by the* Association for Children with Life-Threatening or Terminal Conditions and their Families* (ACT) [6]:

- Category 1: life-threatening diseases in which curative treatment may be feasible but can fail;
- Category 2: diseases whose treatments are long and strenuous and can prolong life and the child participates in normal activities, but premature death is inevitable;
- Category 3: progressive conditions without curative treatment options, where treatment is exclusively palliative;
- Category 4: irreversible but nonprogressive conditions, with complications and likelihood of premature death. CSZV is considered to be included in this group.

However, there were no studies in the researched literature (PubMed, SciELO, and LILACS) that discussed the adoption of palliative care in this population or articles that analyzed data on hospitalizations or search for emergency care services.

The goal of the present study was to determine the frequency and factors associated with hospitalization and emergency care of patients with CSZV, in addition to observing the interventions performed at these moments, analyzing them according to the perspective of palliative care.

## 2. Material and Methods

A cross-sectional observational study with internal comparison group and bidirectional longitudinal component (concurrent and nonconcurrent) involving patients with CSZV was held at the Institute of Integral Medicine Prof. Fernando Figueira (IMIP), which is one of the main centers of reference for children with CSZV and their families, with outpatient follow-up by multidisciplinary team, emergency care, and hospitalizations. The research involved patients born with CSZV as of August 2015 and followed up until October 2017 and data collection occurred between May and October 2017.

Patients with characteristic neurological images on computed tomography (CT) and magnetic resonance (MR) and/or positive IgM serology for Zika virus in cerebrospinal fluid (CSF) were included. A list of patients with diagnostic confirmation was made at the outpatient clinic specialized in CSZV, followed by an analysis of the medical records and a search in the hospital's internal information system.

In the prospective component of the research, the inpatients' companions were informed about the purpose of the study and, upon agreeing to participate, a free informed consent form was requested, and the questionnaire was completed by interview and analysis of medical records.

The variables analyzed in the study involved, among the maternal characteristics, age, occupation, origin, and schooling. Among the variables of the patients evaluated were sex, HC at birth, diagnosis by imaging examination or CSF, emergency care and hospitalization service frequency, age and reason for consultations/hospitalization, indication of intensive care in Intensive Care Unit (ICU), invasive procedures, palliative care approach, length of hospitalization, and reason for leaving the hospital.

Data accuracy was ascertained by double entry of all data obtained in the program Microsoft Excel, exported and compared in order to correct inconsistencies. Epi Info 5.4 and GraphPad Prism 7.0 software were employed for statistical analysis. The association between categorical variables was assessed by calculating odds ratio and Chi-square test or by Fisher's exact test, where relevant. For the numerical variables, the Shapiro-Wilk test was initially applied. The HC at birth was the only variable that presented normal distribution and therefore was analyzed by calculating mean and standard deviation, while for the other variables the median and minimum and maximum measures were calculated. The nonparametric Mann-Whitney test was used to compare numerical variables with non-normal distribution between two groups, while Student's t-test was used to compare variables with normal distribution. A p value less than 0.05 was considered significant.

This project was approved by IMIP's Ethics Committee in Research under protocol no. 54701516.1.0000.5201.

## 3. Results and Discussion

The present study analyzed the characteristics of the patients followed by CSZV and evaluated possible associated factors for a greater number of consultations in the emergency care and hospitalization. In hospitalizations prospectively accompanied by the principal investigator, it was observed whether measures of palliative care were discussed or adopted together with the family.

A total of 145 patients were identified in our specialized clinic. Of these, 74 (51.0%) maintained regular follow-up, 41 (28.2%) were transferred to follow-up clinic near the home city or to other referral centers in the metropolitan region, 5 (3.5%) died, and 25 (17.2%) lost follow-up in this period. The maternal and the patients with CSZV characteristics are presented in Table 1.

Most mothers (58.6%) were housewives and it was observed during the review of records and individual interviews that many mothers who had formal or informal work outside the home before the birth of these children had to leave their previous jobs to dedicate themselves to looking after the children.

The level of maternal schooling was higher than that described in the literature [19], with a minimum of five years of schooling, and none of the mothers were illiterate. The low age of progenitors was consistent with findings in other studies [4, 8], demonstrating the reality of young mothers who need to dedicate special attention to children with different needs [15, 22]. Most mothers were housewives and despite receiving government financial benefit, the costs associated with the care required by these children are quite high, such as transportation, medical care, medications, and rehabilitation [22].

Among the 145 patients, 53.8% were females and 53.1% were from the countryside of the city. The HC at birth was between 22 and 34 cm with a mean of 28.9 cm (standard deviation, SD ± 2.1).

All patients underwent cranial tomography and 110 (75.9%) performed CSF's collection. Most patients (76.6%) had changes in CT considered suggestive of CSZV, but with a negative CSF serology for Zika virus; one had no characteristic CT changes but positive CSF serology and 22.8% had characteristic changes both in the tomography and CSF serology positivity.

**Figure 1:**
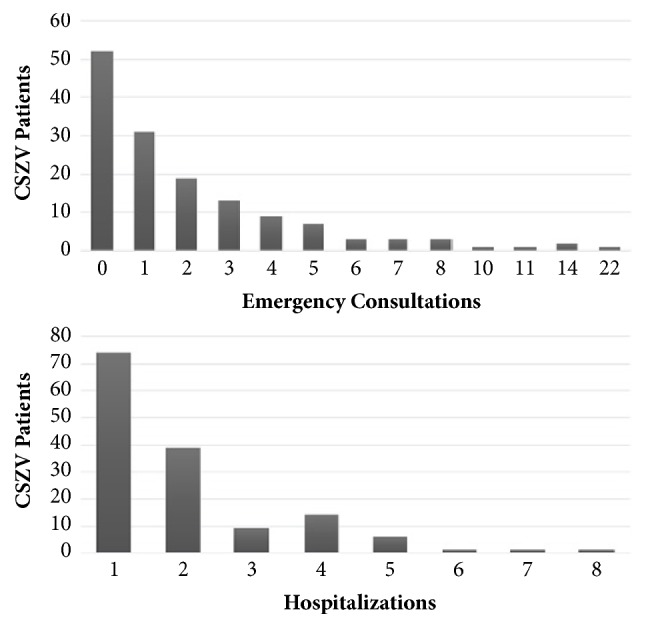
Number of emergency visits and admissions of patients with CZVS up to 25 months of life. IMIP, August 2015 to October 2017.

In the service, 313 consultations regarding patients with CSZV were identified. Among the 92 patients who were consulted in the emergency service between the neonatal period and 20 months of age, the average was 7 months of age and the maximum number of visits per patient was 22 times, as shown in Figure 1. About 1/3 (36.5%) of the children in regular follow-up never needed this type of care.

Despite the lack of data on the occurrence of consultations in other emergency services, it is understood that the high number of consultations in this group is associated with the complications of CSZV such as dysphagia, neurological conditions, and irritability, as described in several studies [13, 19, 23, 24].

It was observed that patients from Metropolitan Region of Recife (MRR) had more consultations in emergency services, which can be attributed to the ease of access to the service, but this finding did not present a statistically significant difference.

In total, 143 hospitalizations were analyzed. The first hospitalization occurred from the neonatal period up to the full 24 months, with an average of 7 months. Half of the patients (51%) never needed to be hospitalized, and the number of hospitalizations per patient ranged from one (26.2%) to eight (0.7%). The main cause of the first hospitalization was a neurological condition associated with CSZV (42.2%) and described in medical records such as microcephaly, hydrocephalus, seizures, or somnolence due to intracranial hypertension [13, 15–18, 23].

Observational studies have shown evolution for hydrocephalus in approximately 41% of patients with CSZV in the first 17 months of life, with an indication of VPS in all of these [24]. The pathophysiology of the evolution of these cases for hydrocephalus is still not well known, but according to some hypotheses due to the damage to the cerebral vascular system, especially in the venous component, there is thrombosis and cerebral venous hypertension since intrauterine growth, persisting after birth, resulting in chronic venous cerebral hypertension [24–26].

The hospitalizations occurred to perform invasive procedures (9.7%) such as VPS implantation, gastrostomy, or orthopedic surgeries due to complications inherent to the basis disease. Respiratory infections accounted for 4.1% of hospitalizations of these children, understood as possible consequences of bronchoaspirations due to neurological disorders and swallowing [13, 15–17]. As observed in other studies, this pattern of complications would be expected for these children; however, the high number of hospitalizations and invasive procedures stands out [13, 15–17].

The duration of hospitalization was between one and 39 days, with an average of four days. Those who were discharged on the same day had been admitted for minor surgical procedures, such as herniorrhaphy or prostatectomy. The discharge from the hospital occurred by improvement (93%), transfer (6.3%), and death (0.7%).

Prolonged admissions, although not frequent, call the attention to the potential for complications, especially in relation to healthcare-associated infections, to the change in family dynamics in this period, compromising the patient and family's quality of life, in addition to the high cost to the health system [22].

Risk factors for inpatient and outpatient emergency's consultation were analyzed and compared to the nonhospitalized and non-urgently consulted groups, both with CSZV. Among the variables studied, no factor was associated with the higher occurrence of hospitalizations or consultations in the emergency room, as it can be observed in Table 2.

It was possible to observe hospitalizations of 13 patients prospectively and the data are shown in Table 3. Of the hospitalized patients, eight were females, the HC of birth was between 28 and 32 cm (average of 29.3 cm and SD ± 1.0), and the age at admission was between 16 and 24 full months (average of 20 months).

Almost all these patients (92.3%) underwent invasive procedures during hospitalization, with the most common being peripheral venous puncture (92.3%), but nasogastric tube (NGT) was also used in 46.1% (six patients), the central venous puncture in 15.4% (two patients), gastrostomy in a patient (7.7%), and oxygen support in 46.1% (six) of patients, ranging from oxygen catheter to orotracheal intubation in an emergency room in one of the patients (7.7%).

It was possible to observe, with emphasis on the analysis of aspects related to palliative care, the high occurrence of invasive procedures. Such procedures provoke pain during its accomplishment and discomfort during the period of use. Moreover, invasive devices can facilitate colonization by nosocomial bacteria and cause local and systemic infections [27]. Therefore, procedures may become useless and an idle cause of suffering if they are not indicated [20].

Functional assessments according to the Lansky scale [19] based on neurological evaluation and accompanying reports were recorded. The largest evaluation was quantified in “40” for two (15.4%) of the patients (participating in quiet activities), four (30.8%) had their functional evaluation in class “30” (in need of assistance even for quiet activity), and the others (53.8%) had a quantified “20” evaluation (with playing entirely limited to very passive activities). This tool is an auxiliary measure in the elaboration of the care plan for these patients [19, 20] and showed the limitation regarding activities of daily living and absence of autonomy of these patients.

Two (15.4%) of the patients had their records requested for a first-time ICU vacancy, both due to acute respiratory failure. After a better understanding by the medical team that assisted them regarding the functionality, characterization of the clinical condition, and association with the basis disease, the request for a vacancy in the ICU was suspended and after conversation and clarification with companions, palliative care measures were indicated: patient number three was submitted to palliative extubation according to precepts of palliative care and airway support was installed with continuous positive pressure; patient number nine had the bladder catheter of delay and central venous access removed; for both the order of non-resuscitation was expressed and symptom control measures were performed (analgesia).

However, most families and caregivers received no approach to palliative care during hospitalizations, despite the basic conditions of the children. It is understood that, as an emerging aspect of pediatric practice, professionals postpone such an approach until the end of life or do not use it [21, 27].

The need to perform surgical interventions and long hospitalizations in ICU is frequent in children born with malformations, which implies thinking about the limits of the therapeutic effort in these cases [27]. According to precepts of palliative care, obstinate therapeutic investments in patients without a cure perspective, such as the cases demonstrated above, should be avoided, but in practice many ICU beds are occupied, sometimes through judicial action, by patients with no possibility of therapeutic control and with underlying diseases that limit survival and interfere greatly in the quality of life, such as CSZV [21, 27]. During the observation of hospitalizations, it was seen that the indication of ICU treatment can be reversed and patients received measures to minimize suffering, avoiding painful interventions, as well as being accompanied by family throughout the hospitalization period, including the evolution to death.

One of these patients died during hospitalization at 19 months of age and the family was present at all times, including death. According to a report from the Secretary of Health of Pernambuco, from the beginning of the appearance of these cases until October 2017, 2403 notifications were made, of which 438 were confirmed as CSZV and 28 (6.4%) deaths were documented in the postneonatal period [28]. According to data from the epidemiological bulletin provided by state agencies, the cases of postneonatal deaths had an average age of 6.2 months of life (ranging from 32 days to 21.9 months) [28].

The other patient with indication of palliative measures received discharge from hospital with written guidelines in summary over the care plan that was drawn up during and after hospitalization. The remaining patients were discharged and there was no report or description of approach in palliative care intended for them.

## 4. Conclusions

Despite the short lifespan of children with CSZV, it could be observed through this study that they were submitted to a high number of emergency room visits, as well as hospitalizations with excessive amounts of invasive procedures. It is understood that the patient with severe malformations already known as those caused by congenital infection by Zika virus could be identified early for care that would allow life without suffering and without any disproportionate invasive methods [27].

## Figures and Tables

**Table 1:** Maternal and patients with CZVS characteristics. Institute of Integral Medicine Prof. Fernando Figueira, August 2015 to October 2017.

**Maternal Characteristics**	N (%)	**Patients with CSZV**	N (%)
		(i) **Female**	78 (53.8)
(i) **Schooling (years)**		(ii) **Head circumference at birth (Z score for age and sex)**	
≤ 4	3 (2.1)	< z -3	101 (69.5)
5-9	44 (30.3)	z-3 to z-2	21 (14.5)
10-11	64 (44.1)	z-2 to z-1	13 (9.0)
≥ 12	10 (6.9)	> z -1	10 (7.0)
No registry	24 (16.6)		
		(iii) **Age (months)**	
		<12 m	25 (17.3)
		12-18 m	39 (26.9)
		>18 m	81 (55.8)
(ii) **From**		(iv) **Consultation in emergency care**	92 (63.5)
MRR^a^	68 (46.9)		
		(v) **Hospitalization**	71 (49.0)
		(a) **Causes of hospitalization**	
(iii) **Occupation**		Neurological Causes	24 (33.8)
Housewives	85 (58.6)	Invasive procedures^b^	14 (19.8)
Formal Work	18 (12.4)	Respiratory infections	6 (8.5)
Informal Work	14 (9.7)	Wheezing	4 (5.6)
Student	6 (4.1)	Dysphagia	1 (1.4)
No data	22 (15.2 )	Others	18 (25.3)
		No registry	4 (5.6)
(iv) **Age (years)**		(b) **Length of hospitalization (days)**	
≤17	15 (10.3)	≤ 1	12 (16.9)
18-24	62 (42.8)	2-7	46 (64.8)
25 - 34 years	56 (38.6)	8 -14	8 (11.3)
≥35 years	11 (7.6)	15 - 30	4 (5.6)
No registry	1 (0.7)	> 30	1 (1.4)

a- Metropolitan Region of Recife

b- Described as invasive procedures: peritoneal ventricle shunt, gastrostomy, herniorrhaphy, prostatectomy.

**Table 2:** Univariate analysis of the possible factors associated with emergency visits and hospitalization in patients with SCZV. Institute of Integral Medicine Prof. Fernando Figueira, August 2015 to October 2017.

Variables	**EMERGENCY CONSULTS**		**PR** ^**b**^ ** (95**%** RI)**	***P***
	Yes N (%)	No N (%)		
	92 (63.4)	53 (36.6)		
Maternal education <10 years	35 (42.2)	12 (31.6)	1.18 (0.89 -1.57)	0.36
Gender (Female)	52 (56.5)	27 (50.9)	1.17 (0.82-1.66)	0.48
Maternal occupation (From home)	64 (77.1)	27 (67.5)	1.41 (0.78-2.57)	0.35
Maternal age (Median)	24	23		0.57
Origin (MRR/Interior)^to^	49/43	19/34	0.67 (0.44 - 1.01)	0.06
Head circumference (Mean)	28.8 4 (± 0.27)	29.01 (± 0.23)	1.30 (0.57 - 1.91)	0.64

	**HOSPITAL ADMISSION**		**PR (95**%** CI)**	***P***
	Yes N (%)	No N (%)		
	71 (49.0)	74 (51.0)		

Maternal education <10 years	24 (38.1)	23 (39.7)	0.97 (0.73-1.29)	0.99
Gender (Male/Female)	36/35	31/43	0.82 (0.58-1.17)	0.36
Maternal occupation (From home)	49 (75.4)	42 (72.4)	1.12 (0.61-2.03)	0.86
Maternal age (Median)	24	24		0.72
Origin (MRR/Interior)	36/35	32/42	0.85 (0.60 to 1.20)	0.46
Head circumference (Mean)	28.97 (± 0.24)	28.93 (± 0.26)	1.16 (0.66 to 1.76)	0.89

a- Metropolitan Region of Recife

b- Statistical analysis: PR: prevalence ratio, p according to Fisher's exact test for categorical variables, Mann Whitney for nonnormal continuous and Student's variables for normal continuous variables (head circumference).

**Table 3:** Characteristics of CZVS patients prospectively observed during hospitalization. Institute of Integral Medicine Prof. Fernando Figueira, May to October 2017.

**Patient**	**Maternal Age** (years)	**Maternal Education** (years)	**Mother Occupation**	**From**	**Sex**	**HC birth** (cm)	**Age** (months )	**Diagnosis** ^**a**^	**Duration** (days)	**Device** ^**d**^	**ICU Indic (Y/N)**	**CCPP (Y/N)**	**Output**	**Scale of Lansky**
1	19	10	Housewife	MRR	M	29	20	Respiratory Infection	4	NGT, PVP, Catheter O2	N	N	Discharge	30

2	21	11	Housewife	MRR	M	30	22	Neurological Cause	10	GTT, PVP, Venturi M.	N	N	Discharge	30

3	-	-	Housewife	Interior	M	29	16	Respiratory infection	12	NGT, OTT, PVP, CPAP, Venturi M.	Y	Y	Discharge	20

4	37	12	Housewife	Interior	M	28.5	17	Neurological Cause	1	PVP	N	N	Transf	20

5	21	11	Housewife	MRR	F	30	20	Other^b^	6	-	N	N	Discharge	20

6	18	9	Housewife	MRR	F	28	22	Wheezing	2	PVP	N	N	Discharge	40

7	33	12	Housewife	MRR	F	29	20	Other^c^	4	PVP	N	N	Discharge	30-20

8	31	12	Housewife	MRR	F	30	23	Respiratory infection	2	NGT, PVP, Venturi M.	N	N	Discharge	20

9	23	12	Housewife	Interior	F	29	19	Respiratory infection	10	NGT, DVC, PVP, CVP, CPAP, Venturi M.	Y	Y	Death	20

10	16	8	Housewife	Interior	F	32	20	Respiratory Infection	49	NGT, VPS, PVP, CVP, Venturi M.	N	N	Discharge	30

11	20	12	Housewife	Interior	M	29	21	Neurological Cause	3	NGT, VPS, PVP	N	N	Discharge	20

12	21	10	Housewife	MRR	F	30	24	Resp Infec	3	PVP	N	N	Discharge	40

13	35	9	Housewife	MRR	M	28	21	Resp Infec	5	PVP	N	N	Discharge	30

a- Diagnoses were divided into categories: respiratory infections (upper and lower airways); neurological causes (epileptic seizures, irritability, hydrocephalus); wheezing; others

b- Other: conjunctivitis

c- Other: fever without localized signs

d- Abbreviations of devices: nasogastric tube (NGT), peripheral venous punction (PVP), central venous punction (CVP), gastrostomy (GTT), orotracheal tube (OTT), Continuous Positive Airway Pressure (CPAP), Venturi mask (Venturi M.), delay vesical catheter (DVC), ventriculoperitoneal shunt (VPS)

e- Acronyms/Abbreviations: Metropolitan Region of Recife (MRR), transference (Transf), male/female (M/F), yes/no (Y N), indications of intensive care unit (ICU Indic), palliative care (CCPP).

## Data Availability

The analysis was performed using the medical records and a search in the hospital's internal information system. Regarding the prospective component of the research, the instrument of data collection was completed by interview and analysis of medical records.

## Supplementary Materials

Supplementary Materials The supplementary files include the “Data Collection Instrument” containing all the information used in this research and “Free and Enlightened Consent Term” signed by the parents or guardians of the children who participated in the study.

## References

[1] Ministry of Health, Secretariat of Health Surveillance, Department of Surveillance of Communicable Diseases, "Surveillance protocol and response to the occurrence of microcephaly related to zika virus infection," Brasília, 2015, [access on Jan. 21. 2017], http://www.saude.gov.br/svs

[2] WHO (US) Birth defects surveillance: a manual for program managers. http://www.who.int/iris/handle/10665/110223.

[3] Teixeira M. G., da Conceição N. Costa M., de Oliveira W. K., Nunes M. L., Rodrigues L. C. (2016). The epidemic of zika virus–related microcephaly in brazil: detection, control, etiology, and future scenarios. *American Journal of Public Health*.

[4] Iglesias S., Zollner A. C. (2016). Pediatric palliative care. *Residência Pediátrica*.

[5] Carvalho R. T., Parsons H. A. (2012). *org. Manual of Palliative Care of ANCP*.

[6] Chambers L., Dodd W., McCulloch R., McNamara-Goodger K., Thompson A. A Guide to the Development of Children's Palliative Care Services. http://www.rcpcf.ru/Files/pdf/ACT.

[7] Ministry of Health, Secretariat of Health Surveillance, Secretariat of Health Care, "Integrated guidelines for surveillance and health care within the Public Health Emergency of National Importance: procedures for monitoring changes in growth and development from gestation to the first childhood, related to the infection by zika virus and other infectious etiologies within the operational capacity of SUS," Brasília, 2017

[8] Lopez-Camelo J. S., Orioli I. M., Castilla E. Document ECLAMC Final: summary and conclusions of Documents 1-5.

[9] Pernambuco, State Secretariat of Health of Pernambuco, Executive Secretariat of Health Surveillance, "Clinical and Epidemiological Protocol for investigation of microcephaly cases in the state of Pernambuco," Version No. 01, Pernambuco, 2015

[10] Pernambuco, State Secretariat of Health of Pernambuco, Executive Secretariat of Health Surveillance, "Clinical and Epidemiological Protocol for investigation of cases of microcephaly in the state of Pernambuco," Version No. 02, Pernambuco, 2015

[11] Araújo T. V. B., Rodrigues L. C., Ximenes R. A. A., Miranda-Filho D. B., Montarroyos U. R., Melo A. P. L. (2018). Association between microcephaly, zika virus infection, and other risk factors in Brazil: final report of a case-control study. *Lancet Infectious Diseases*.

[12] Schaub B., Vouga M., Najioullah F. (2017). Analysis of blood from Zika virus-infected fetuses: a prospective case series. *The Lancet Infectious Diseases*.

[13] Possas C., Brasil P., Marzochi M. C. (2017). Zika puzzle in Brazil: peculiar conditions of viral introduction and dissemination - A Review. *Memórias do Instituto Oswaldo Cruz*.

[14] Oliveira Melo A. S., Malinger G., Ximenes R., Szejnfeld P. O., Alves Sampaio S., Bispo De Filippis A. M. (2016). Zika virus intrauterine infection causes fetal brain abnormality and microcephaly: Tip of the iceberg?. *Ultrasound in Obstetrics & Gynecology*.

[15] Pan-American Health Organization Epidemiological Alert: Neurological syndrome, congenital malformations and zika virus infection: Implications for public health in the Americas. http://www.reliefweb.int/report/world/epidemiological-alert-neurological-syndromecongenital-malformations-and-zika-virus.

[16] Neta T. J. C., Fernandes A. S., Furtado G. (2016). Actions developed at the Institute of Integrale Medicine Prof. Fernando Figueira for coping with microcephaly by zika virus. *Revista Brasileira de Saúde Materno Infantil*.

[17] Souza A. S. R., Cordeiro M. T., Meneses J. A., Honorato A., Junior E. A., Castanha P. M. S. (2016). Clinical and laboratory diagnosis of congenital zika virus and unilateral diaphragmatic paralysis: the report of a case. *Revista Brasileira de Saúde Materno Infantil*.

[18] del Campo M., Feitosa I. M., Ribeiro E. M. (2017). The phenotypic spectrum of congenital Zika syndrome. *American Journal of Medical Genetics Part A*.

[19] Alves L. V., Cruz D. D., Linden A. M. (2016). Epileptic seizures in children with congenital Zika virus syndrome. *Revista Brasileira de Saúde Materno Infantil*.

[20] Botelho A. C., Neri L. V., Silva M. Q. (2016). Presumed congenital infection by Zika virus: findings on psychomotor development - a case report. *Revista Brasileira de Saúde Materno Infantil*.

[21] Alvino A. C., Mello L. R., Oliveira J. d. (2016). Association of arthrogryposis in neonates with microcephaly due to Zika virus - a case serie. *Revista Brasileira de Saúde Materno Infantil*.

[22] Forgotten and unprotected - The impact of the z ika virus on girls and women in northeastern Brazil.

[23] Di Cavalcanti D., Alves L. V., Furtado G. J. (2017). Echocardiographic findings in infants with presumed congenital Zika syndrome: Retrospective case series study. *PLoS ONE*.

[24] Petribu N. C., Aragao M. D., van Der Linden V. (2017). Follow-up brain imaging of 37 children with congenital Zika syndrome: case series study. *BMJ*.

[25] Aragao M. F. V. V., Holanda A. C., Brainer-Lima A. M. (2017). Nonmicrocephalic infants with congenital zika syndrome suspected only after neuroimaging evaluation compared with those with microcephaly at birth and postnatally: How large is the Zika virus "iceberg"?. *American Journal of Neuroradiology*.

[26] França G. V. A., Schuler-Faccini L., Oliveira W. K. (2016). Congenital Zika virus syndrome in Brazil: a case series of the first 1501 livebirths with complete investigation. *The Lancet*.

[27] Chanes I. R., Monsores N. (2016). A bioethical and sanitary reflection on the side effects of the zika virus epidemic: an integrative review on euthanasia/orthanasia in cases of fetal anomalies. *Cadernos Ibero-Americanos de Direito Sanitário*.

[28] (2017). Congenital syndrome related to Zika virus infection. *Technical Report*.

