# Supplementary material for: Palliative Care in Congenital Syndrome of the Zika Virus Associated with Hospitalization and Emergency Consultation: Palliative Care and Congenital Syndrome of Zika
Source: J Trop Med. 2018 Oct 11;2018:1025193. doi: 10.1155/2018/1025193 (PMC6201378; doi:10.1155/2018/1025193)
Supplement: Supplementary Materials — The supplementary files include the “Data Collection Instrument” containing all the information used in this research and “Free and Enlightened Consent Term” signed by the parents or guardians of the children who participated in the study. [file 1025193.f1.docx]

**DATA COLLECTION INSTRUMENT**

**Registration: Name:**

City of origin:

**Maternal data**

Mother's name:

Maternal age (at birth): Maternal education (years of study since literacy):

Maternal occupation: 1. From home 2. Informal worker 3. Formal worker 4. Student 5. Unemployed 6. Retired 7. Other If formal or informal worker, what job? (to describe):

**Patient data:**

D.N .: / / Sex: 1.Male 2.Female PC at birth:

Primary Caregiver:

Inclusion Criteria: 1. CT 2. CSF 3. Both

Follow-up at IMIP: 1.Yes 2.No 3.Does not know / Not informed Where:

Internment / current care:

Where: 1. Infirmary 2. Emergency care Number of hospitalizations:

Date of hospitalization / care: / /

Diagnostics in high order of relevance: 1.Convulsion 2. Diff. Swallowing 3. Respiratory infections 4.Irritability 5.Vomiting 6.Wheezing 7.Other (describe):

| **Impatients’ Number** | | Yes | No | Start Date | End Date | P.S. |
| --- | --- | --- | --- | --- | --- | --- |
| Special diet | |  |  |  |  |  |
| Fasting | |  |  |  |  |  |
| Hydration | |  |  |  |  |  |
| Analgesia | |  |  |  |  |  |
| Sedation | |  |  |  |  |  |
| Anti-thermical | |  |  |  |  |  |
| Antibiotic | |  |  |  |  |  |
| Anticonvulsants (abortion of the crisis) | |  |  |  |  |  |
| Gastric protector | |  |  |  |  |  |
| Pro-kinetic | |  |  |  |  |  |
| Anti-emetic | |  |  |  |  |  |
| Corticoid | |  |  |  |  |  |
| Bronchodilators | |  |  |  |  |  |
| Others: |  |  | |  |  |  |
| Others: |  |  | |  |  |  |

| Exams | Number | Exams | Number | Exams | Number |
| --- | --- | --- | --- | --- | --- |
| Blood count |  | Coagulogram |  | NMR |  |
| Ionogram |  | CPR/ESR |  | US |  |
| Renal function |  | Urine sumarize |  | HDE |  |
| Albumin/RNI |  | Culturs (describe wich place) |  | EEG |  |
| Hepatic enzymes |  | CT |  | Radiography |  |
| Other |  | Others |  | EED |  |

Physical exam data

Wheight: Lenght: CP:

**Current Device Use**

|  | Yes | No | Start date | End date | Number | P.S. |
| --- | --- | --- | --- | --- | --- | --- |
| Nasogastric tube |  |  |  |  |  |  |
| Gastrostomy |  |  |  |  |  |  |
| Bypass shunt |  |  |  |  |  |  |
| Pleural drainage |  |  |  |  |  |  |
| Delayed bladder catheter |  |  |  |  |  |  |
| Peripheral access |  |  |  |  |  |  |
| Central venous access |  |  |  |  |  |  |
| Orotracheal tube |  |  |  |  |  |  |
| Nasal CPAP |  |  |  |  |  |  |
| Venturi Mask |  |  |  |  |  |  |
| Others: |  |  |  |  |  |  |

**Previous hospitalizations without discharge summary**

1. Yes 2. No How many? Wich service?

**Surgeries performed and age at the time of the procedure:**

|  | Yes | No | Age | P.S. |
| --- | --- | --- | --- | --- |
| Orthopedic |  |  |  |  |
| Anti-reflux |  |  |  |  |
| Corrections of anatomical defects |  |  |  |  |
| Others: |  |  |  |  |

**Medicines for continuous use and others at home:**

|  | Yes | No | Tipe | Way | P.S. |
| --- | --- | --- | --- | --- | --- |
| Special diet |  |  |  |  |  |
| Glasses |  |  |  |  |  |
| Hearing orthoses |  |  |  |  |  |
| Orthopedic orthoses |  |  |  |  |  |
| Anticonvulsants |  |  |  |  |  |
| Gastric protetor |  |  |  |  |  |
| Pro-kinetic |  |  |  |  |  |
| Corticoid |  |  |  |  |  |
| Bronchodilators |  |  |  |  |  |
| Others: |  |  |  |  |  |
| Others: |  |  |  |  |  |

**Complications and / or adverse events:**

|  | Yes | No | P.S. |
| --- | --- | --- | --- |
| IRAS |  |  |  |
| IPCS |  |  |  |
| Pneumon |  |  |  |
| Surgeric place |  |  |  |
| Urinary tract |  |  |  |
| Venous access related |  |  |  |
| Meningoencephalitis by shunt |  |  |  |
| Shunt’s obstructions |  |  |  |
| Leakage or displacement of gastrostomy |  |  |  |
| Others: |  |  |  |
| Others: |  |  |  |

**Specialties in monitoring:**

|  | Yes | No | Dont know | P.S. |
| --- | --- | --- | --- | --- |
| Pediatrics |  |  |  |  |
| Neurology |  |  |  |  |
| Gastroenterology |  |  |  |  |
| Pneumology |  |  |  |  |
| Orthopedy |  |  |  |  |
| Ophthalmology |  |  |  |  |
| Speech therapy |  |  |  |  |
| Phisiotherapy |  |  |  |  |
| Occupational therapy |  |  |  |  |
| Nutrition |  |  |  |  |
| Psychology |  |  |  |  |
| Otolaryngology |  |  |  |  |
| Others: |  |  |  |  |
| Others: |  |  |  |  |

**Indication / Request for vacancy in ICU**: 1.Yes 2.No 3.Does not know / Not informed

Reason: 1. Change in level of consciousness 2. Respiratory insufficiency 3. Hemodynamic instability 4. Other:

Admission to the ICU: 1.Yes 2.No Date of admission ICU: / / Date of ICU discharge: / /

**Palliative care:** -Family approach: 1. Yes 2. No -Extubation: 1. Yes 2. No

                                      - Order not to resuscitate: 1. Yes 2. No - Withdrawal of devices: 1. Yes 2. No

- Pain control: 1. Yes 2. No - Other (describe):

**Date of departure**: / /

Output: 1.Home 2. Discharge 3.Transfer If transfer, where:

Place of death: 1. House 2. Hospital 3. Others:

Comments:

FREE AND ENLIGHTENED CONSENT TERM

Research: “Palliative Care in Congenital Syndrome of the Zika virus associated with hospitalization and emergency consultation.”

Dear _______________________________________________ (**father’s or mother’s name or another responsible),** father/mother/responsible of____________________________________________.

My name is Aline Maria de Oliveira Rocha and I am a resident physician in Pediatrics and researcher of this project. We are doing a research on the health problems in children who were born with Zika's Congenital Syndrome virus. This research intends to know better the symptoms and complications more prevalent of these children so that these patients can be better evaluated, treated and accompanied.

Your participation and that of your child would provide important information to health professionals about the most common problems that have led your child to the emergency room and/or hospital and how to reduce problems and / or improve their development. Therefore, I ask your permission for your child to participate in the research. We request you to allow clinical observation and annotation of important points during the research period.

Your child's treatment will follow the IMIP routine, with staffing, interviews and guidance. Although we can not immediately deliver the study results, we can send it to your home as soon as we have the outcome if you wish. You should feel completely free to participate in the study. Whether or not you participate in this study will make no difference in the treatment of your child, and you have every right to ask to be excluded from the study at any time you deem necessary. This will not cause any restrictions on the treatment of your child nor will it cost you money.

People will not know your child's name or yours, and the data from this survey will help you better understand your child's illness and will be shown in journals and science classes. If it is necessary to show the images of exams already carried out, it will be guaranteed that the identity of the child or his / her family will not be revealed.

We request the authorization to consult additional information in the medical records and we guarantee that no information that can identify your child or your family will be revealed.

If you have any questions regarding the research, you may contact me at (83) 9 88010605 or with responsible researchers at (81) 2122-4147. You may also contact IMIP's Human Research Ethics Committee (CEP-IMIP), which aims to defend the interests of the participants, respecting their rights and contributing to the development of the research, provided they comply with ethical conduct. CEP-IMIP is located at Rua dos Coelhos, 300, Boa Vista. IMIP Research Directorate, Orlando Onofre Administrative Building, 1st Floor tel: 2122-4756 Email: comitedeetica@imip.org.br The CEP / IMIP works from Monday to Friday, at the following times: 07:00 a.m. to 11:30 p.m. (morning) and 13:30 to 16:00 (afternoon).

Recife, ________ of _____________.

___________________________________ ___________________________

Father, mother or responsible First attestant

___________________________________ ___________________________

Father, mother or responsible Second attestant
